# Aflatoxin M_1_ levels in different marketed milk products in Nairobi, Kenya

**DOI:** 10.1007/s12550-018-0323-4

**Published:** 2018-08-14

**Authors:** Johanna Frida Lindahl, I. N. Kagera, D. Grace

**Affiliations:** 1 International Livestock Research Institute, PO Box 30709, Nairobi, 00100 Kenya; 2 Department of Medical Biochemistry and Microbiology, Uppsala University, Box 582, 75123 Uppsala, Sweden; 3 Department of Clinical Sciences, Swedish University of Agricultural Sciences, PO Box 7054, 75007 Uppsala, Uppsala Sweden; 4 Department of Food Science and Technology, Jomo Kenyatta University of Agriculture and Technology, PO Box 62, Nairobi, 000-00200 Kenya

**Keywords:** Mycotoxins, Food safety, East Africa, Dairy, Chemical hazard

## Abstract

Milk is an important source of energy and nutrients, especially for children, and in Kenya, milk consumption is higher than other countries in the region. One major concern with milk is the risks of chemical contaminants, and reports of high levels of aflatoxin M_1_ (AFM_1_) in milk in Kenya has been causing public health concerns. This study collected marketed milk products every month during 1 year, just as a consumer would purchase them from retailers and traders in a low-income area, and a major supermarket in a middle/high-income area. In total, 291 sampled milk products (raw, pasteurised, UHT milk, yoghurt and lala) were collected and analysed for AFM_1_ using a commercial ELISA kit. More than 50% of the samples exceeded 50 ng/kg (the level allowed in the EU), but only three samples exceeded 500 ng/kg (the level allowed in the USA). Geometric mean AFM_1_ level was 61.9 ng/kg in the 135 samples from the low-income area while it was 36.1 ng/kg in the 156 from the higher income area (*p* < 0.001). The levels varied significantly depending on the time of year, with lowest levels of milk in January. There were also differences between manufacturers and products, with UHT milk having lower levels. There was no difference depending on the price for all dairy products, but when only including milk, higher price was associated with lower levels of AFM_1_. In conclusion, this study shows that milk purchased by a consumer is likely to contain AFM_1_ above 50 ng/kg, and that further research is needed to find ways to mitigate AFM_1_ contamination through working with farmers and milk processors both in the formal and informal sectors.

## Introduction

Milk is an important source of both micro- and macronutrients and is often targeted to children and pregnant women, but children in many low- and middle-income countries often consume too little (Dror and Allen 2011). Kenya has some of the highest milk consumption in Africa: various studies estimate that Kenyans consume between 50 and 150 l of milk per person each year, and consumption is growing rapidly (Smallholder Dairy Project 2004; Bosire et al. 2017).

Milk is an excellent substrate for bacterial growth, originating either from the cow, the environment, or the milk handlers; chemical contaminants may enter the milk through the feed or treatments of the cow or through later accidental or deliberate contamination. A major cause of concern is the contamination of milk with aflatoxin M_1_ (AFM_1_), which can occur where dairy animals eat contaminated feeds. Aflatoxins are mycotoxins produced by fungi, mainly *Aspergillus flavus*, growing on crops or food products. When animals ingest the feed containing AFB_1_, it is metabolised intoAFM_1_, which is excreted in urine as well as in the milk of lactating females. While the rate of carry over may vary between cows, depending on factors such as the productivity, no studies have focused on the situation in East Africa. Studies elsewhere indicate that 1–7% of the aflatoxin B_1_ (AFB_1_) ingested by cows may be carried over as AFM_1_ into the milk (Masoero et al. 2007; Fink-Gremmels 2008).

The different aflatoxins are all toxic and carcinogenic, with AFB_1_and AFM_1_being class 1 carcinogens, and AFM_1_ being less carcinogenic in animals trials (Cullen et al. 1987; IARC 2002). Good evidence exists for a link between hepatocellular carcinoma and aflatoxins in humans (Liu and Wu 2010), while in livestock, there is strong evidence that aflatoxins cause immunosuppression and depress growth (Atherstone et al. 2016). Some studies have indicated an association between aflatoxin exposure, or aflatoxin biomarker levels, and stunting in children, but other studies have failed to find an association (Khlangwiset et al. 2011). Previously published work in Kenya has shown an association between exposure to AFM_1_ in milk and reduced growth in children from low-income areas in Nairobi, where 41% of children were stunted (Kiarie et al. 2016). That study detected AFM_1_ in all milk samples from households tested, and milk contributed daily a median of 3.7 ng AFM_1_ to the children, with some children consuming more than 100-ng AFM_1_ from milk per day.

This study was conducted to shed light on the levels of AFM_1_; a typical Nairobi milk consumer may be exposed to in a high/middle-income and low-income area, respectively.

## Material and methods

### Sample collections

Marketed milk and milk products were purchased during the second week of every month for 1 year, starting from November 2013 to October 2014. According to the objectives of our study, milk was purchased as a consumer would purchase it. One supermarket was purposively chosen, belonging to one of the major Kenyan supermarket chains, located in a shopping centre in a middle- to high-income area in Westlands, Nairobi. In Dagoretti, a low-income area, retailers and traders were selected by the data collector travelling to the major junction and, from there, takes different directions each time and collects milk from as many retailers as needed until the target number of samples were reached. Thus, all retailers were conveniently sampled from a walking distance of the major junction.

At each collection date, six samples were purchased of each of the following categories; raw milk, fresh pasteurised milk, ultra-high temperature (UHT)-treated milk and fermented milk products (including yoghurt and lala (maziwa lala/mala; a locally fermented milk). The raw milk samples and six of the other products were purchased from retailers and milk traders in Dagoretti, and the rest of the samples were purchased in the supermarket in Westlands.

All samples purchased the same day were unique and not pooled; the raw milk samples were all collected from different milk bars/kiosks, and the packaged milk products were all from different manufacturers or different products. Raw milk was purchased in units of 500 ml, and packaged milk products were purchased in units of at least 100 ml. The purchased milk was handled as normal consumers would be expected to handle the products and carried in the shopping bag provided by the seller to the lab within 2 h. In the lab, samples were aliquoted into two plastic tubes of 50 ml each and stored at – 20 °C until the time of analysis which was within 3 months after date of purchase.

Data was collected for each sample on the quantity purchased and the price it was purchased at, on the production and expiry dates if provided, the processor, package and storage type, if the samples were kept cold or not in the place of purchase or if the products were labelled with the approval logo of the Kenyan Bureau of Standards (KEBS). When aliquoting, it was also noted if the product had abnormal smell or appearance. Production date for raw and boiled milk samples was assumed to be the same day of sampling.

### Laboratory analyses

Aflatoxin M_1_ was detected using a commercial enzyme-linked immunosorbent assay (ELISA) (Helica Biosystems Inc., Santa Ana, CA, USA, catalogue no. 961AFLM01M-96) following the manufacturer’s instructions, summarised in brief.

Homogenised milk was used directly in the assay. Mixed unprocessed raw fatty milk, as well as samples of yoghurt and lala, was refrigerated for 1 h and centrifuged at 2000*g* for 5 min to induce separation of upper fatty layer. The upper fatty layer was removed, and the lower plasma layer of the milk was used in the assay.

Standards and samples (200 μl) were aliquoted to the pre-coated plates in duplicates. No additional reference samples were used. After 2 h of incubation and washing, 100 μl of conjugate was added. After a 15-min incubation and washing, 100 μl of enzyme substrate was added to each well and incubated for 15 min before adding 100 μl of stop reaction. The optical density of each microwell was read using a microplate reader at 450 nm, and the level of AFM_1_ in each well was calculated using a logarithmic standard curve (requiring an *R*^2^ value of above 95%), and the average of the duplicates was used as results. The ELISA used had a lower limit of detection of 2 ng/kg according to the manufacturer, and this was assumed to be accurate. Samples exceeding the highest standard (100 ng/kg) were diluted and re-tested in duplicates, but not repeated. The methods have been described previously (Kiarie et al. 2016; Kirino et al. 2016; Senerwa et al. 2016). Spiking of samples has been conducted to test the accuracy of this kit in another project (Berhanu et al. under preparation) and show recovery rates being between 70 and 156%, with negative samples not exceeding 4 ppt (Table 1).

**Table 1:** Recovery rates of spiked samples using the commercial enzyme-linked immunosorbent assay (ELISA) (Helica Biosystems Inc., Santa Ana, CA, USA, catalogue no. 961AFLM01M-96) (Berhanu et al. under preparation)

Concentration in the spiked sample	Concentration received using the ELISA	Recovery rate (%)
0	2.1	
0	2.4	
0	3.5	
20	20.5	102
20	27.6	138
20	21.8	109
20	19.5	97
20	21.0	105
25	25.8	103
25	17.5	70
50	56.5	113
50	77.8	156
50	49.9	100

### Statistical analyses

Data was entered into excel. Analyses were done using STATA 14.0. For statistical analysis, the level of samples with a result under the level of detection (5 samples) was substituted with 1 ng/kg (half the level of detection). Manufacturers from which there were less than 10 samples were all classed into one category, which created a total of six categories: four dairy-producing companies (company A–D), farmers and other manufacturers. For pasteurised and UHT milk samples where production date was not available, the production was assumed to be the month before the expiry date, since this was most commonly found with the samples that had production date stated.

Univariable analyses were done using chi-square, Student’s *t* test and regression. Multivariable linear regression was done with logarithmic AFM_1_ levels as the dependent variable, using mixed procedure with area as the random variable. Fractional polynomials with two dimensions were used for month of sampling, with the months adjusted to set May to be the first month in order to create a cyclic pattern. Since the type of product was correlated with the treatment, only treatment was included in the model.

## Results

In total, 291 milk samples were collected, 135 were from the low-income area (Dagoretti) and 156 from the higher income area (Westlands). All packaged products had a KEBS stamp, except one that was approved by the Ugandan Bureau of Standards. No products were judged abnormal as to appearance. Overall, more than 50% of the samples exceeded EU legal limit of 50 ng/kg AFM_1_, but only three samples exceeded 500 ng/kg—two milk samples from Dagoretti and one yoghurt sample from Westlands (Table 2). Geometric mean AFM_1_ level was 61.9 ng/kg in Dagoretti, while it was 36.1 ng/kg in Westlands (*p* < 0.001).

**Table 2:** Aflatoxin M_1_ levels in different milk products purchased from two different parts, Dagoretti and Westlands, of Nairobi, Kenya

	Number of samples	Mean aflatoxin M_1_ levels	Standard deviation	Min	Max	Above 50 ng/kg (%)	Above 500 ng/kg
Dagoretti	135	106.1	140	< LOD	1100	89 (65.9)	2
Lala	8	111	121	10	340	5 (62.5)	0
Milk	110	107	149	< LOD	1100	71 (64.5)	0
Boiled	13	46	23	14	88	5 (38.5)	0
Pasteurised	18	126	189	< LOD	740	13 (72.2)	1
Raw	62	131	165	< LOD	1100	46 (74.2)	1
UHT	17	46	24	7.3	84	7 (41.2)	0
Yoghurt	17	96	75	26	270	13 (76.5)	0
Westlands	156	66	107	< LOD	1100	62 (39.7)	1
Lala	27	48	35	12	160	8 (29.6)	0
Milk	108	57	72	< LOD	470	42 (38.9)	0
Pasteurised	53	55	34	7.6	210	26 (49.1)	0
UHT	55	58	95	< LOD	470	16 (29.1)	0
Yoghurt	21	134	232	17	1100	12 (57.1)	1
Total	291	84	125	< LOD	1100	151 (51.9)	3

All lala and yoghurt products were pasteurised

*LOD* Limit of detection (2 ng/kg)

There were differences in the distribution of different brands purchased in Westlands and Dagoretti. In Dagoretti, more than half of the samples from company C (the cheapest brand (Table 3)) were purchased. Linear regression showed no association between the price of the milk product and the AFM_1_ levels, either as logarithmic or actual values when including all samples. However, when only studying milk samples (raw, boiled, pasteurised or boiled), there was a significant (*p* < 0.001) inverse relationship between price and AFM_1_ levels (both as logarithmic and actual values). The cheapest milk was found in Dagoretti, where boiled milk could be purchased from 45 Kenyan shilling (KES) per litre and raw milk from 50 KES per litre (1 KES = 0.01 USD in 2014). The most expensive products were yoghurt purchased in quantities of less than 200 ml, where the litre price exceeded 600 KES per litre.

**Table 3:** Aflatoxin M_1_ levels in milk samples of different origins purchased in Nairobi, Kenya, and the price (Kenyan shilling (KES) (1 KES = 0.01 USD in 2014))

Producer	Number	Mean price KES/litre (range)	Mean aflatoxin M_1_ levels (ng/kg)	25% quartile	Median	75% quartile	Geometric mean
Farmers	75	65 (45–110)	117	39	79	130	65.6^a^
Company A	74	155 (80–610)	57.0	33	44	69	46.4
Company B	12	101 (90–120)	297	120	270	440	227
Company C	51	128 (60–233)	37.2	9.1	29	51	22.7^b^
Company D	37	125 (86–233)	38.9	14	31	59	23.7^b^
Others	42	176 (76–660)	111	35	68	93	68.0^a^

Geometric means with the same superscript were not significantly different

*LOD* Limit of detection (2 ng/kg)

**Fig. 1:**
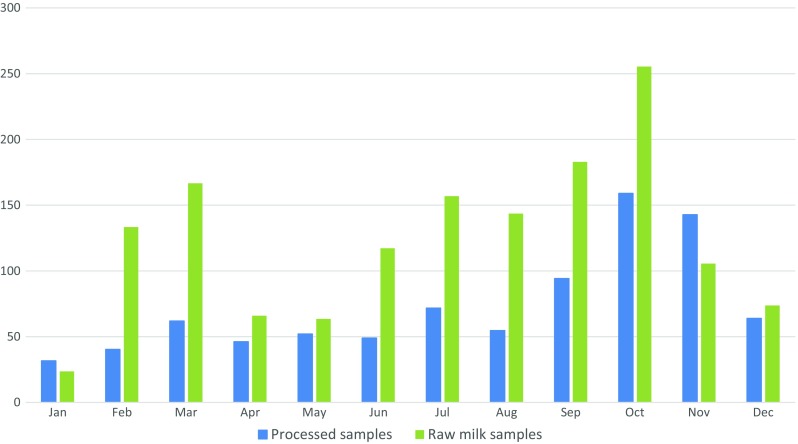
**a** The average aflatoxin M_1_ (AFM_1_) levels depending on the month of production of the milk sampled in Nairobi, Kenya. **b** The average AFM_1_ levels depending on the month of sampling, for both all milk samples and for the raw milk samples, where the raw milk samples are always produced in the same month as the sampling

There were significant differences in AFM_1_ levels depending on the producer of the milk (Table 3). Company C had significantly higher levels than the others, and two companies had levels significantly lower than the rest. The levels of AFM_1_ were not uniformly distributed, but the average level peaked in milk sampled between September and November, with highest levels in October (the start of the short rain season) (Fig. 1a). A small peak was also observed in March (the start of the long rain seasons). The same trend was seen when studying production dates (Fig. 1b), and this trend was more pronounced when including only the raw milk samples.

The multivariable regression model (Table 4) showed that both treatment of milk and the month of sampling affected the AFM_1_ levels, with UHT milk having the lowest levels; however, the model had a low fit and did not produce normally distributed residuals.

**Table 4:** The results of a multivariable model for the effects of month and treatment of milk on aflatoxin M_1_ level (log transformed)

Parameter	Coefficient	*P*
Seasonal effect		
Month dimension 1 (cubic)	2.2	0.03
Month dimension 2 (square root)	− 2.0	0.01
Pasteurised	Reference	
UHT	− 0.7	< 0.001
Raw	0.04	0.8

## Discussion

This project focused on the level of AFM_1_ in urban-marketed milk in Kenya, with the approach of buying milk as consumers in low-income and middle/high-income areas would do it. We observed levels as high as 1000 ng/kg in milk products, which is higher than what has been described by some studies earlier (Kang’ethe and Lang’a 2009) but lower than the maximum levels found in another recent study of raw milk (Sirma et al. 2016). The levels of AFM_1_ in samples from raw milk traders in Dagoretti has been reported before (Kirino et al. 2016), with results from raw milk samples were similar, with 55% of samples exceeding 50 ng/kg.

This study showed a variation over the year as to the levels detected, but since only 1 year was included, it is difficult to draw conclusions on seasonality. Previous research has shown that there are seasonal variations in AFM_1_ levels which may depend on availability of fresh grass and the time of storage of feeds from the last harvest, and in Kenya, higher aflatoxin levels in feeds have been observed during the rainy season compared to the dry season (Xiong et al. 2013; Bilandžić et al. 2014; Senerwa et al. 2016). Similarly, early studies in Kenya indicated more human exposure during the rainy season (De Vries et al. 1989). Further studies into these dynamics may be needed in order to better direct mitigation strategies.

Our study found significant differences between companies vending milk products, which could indicate that the companies procure milk from different sources and have more or less stringent control mechanisms. This warrants further studies exploring potential explanations for these differences. Unlike observations from maize flour, where more expensive maize flour had lower levels of aflatoxins (Hoffmann and Moser 2017), our study showed no significant association between the price of the dairy products and the levels of AFM_1_ when all products were included, but when yoghurt and lala were excluded, there was an association with more expensive milk having less AFM_1_, which is also to be expected since we found higher levels in raw milk samples, and these were also the cheapest.

Previous work has shown that milk consumers state that they would be willing to pay more for aflatoxin-safe milk (Walke et al. 2014; Mtimet et al. 2015), and use of certification of milk products as safe has been suggested as one approach to aflatoxin mitigation, whereby the premium for certification would incentivise efforts in control (Johnson et al. 2015), but the authors also note the potential risk that a differentiated market could be anti-poor. However, the differences between different milk producers indicate a need to conduct further research in collaboration with milk processors to identify the practices behind the differences between processors, including finding how the different processors source their milk and work with quality assurance at farm level.

Even though AFM_1_ does not disappear by pasteurisation, there were significantly lower levels in UHT milk, and similar results have been found by other studies (Iha et al. 2013; Zheng et al. 2013). This might be explained by the timing of production, since UHT is more likely to be produced when milk is surplus (towards the end of the long and short rains) and from certain geographical regions, but this would not be in accordance with the feeds having higher AFB_1_ levels during rainy season as has been reported before (Senerwa et al. 2016). Although aflatoxins are considered heat-stable, there are studies indicating that there still be an impact from heat treatments and other methods of processing (Purchase et al. 1972; Scott 1984; Kabak 2009; Fernandes et al. 2012), but results have been conflicting, and further research is warranted.

The public health importance of AFM_1_ levels in milk has never been fully elucidated. In animal trials, AFM_1_ has been shown to be much less carcinogenic than AFB_1_ (Cullen et al. 1987; JECFA 2001). Although studies have found an association between stunting and aflatoxins (Khlangwiset et al. 2011), evidence of causation is still lacking. In Nairobi, an association between AFM_1_ exposure and lower height-for-age scores has been observed (Kiarie et al. 2016), and similarly, a study in Iran showed that infants of mothers which had AFM_1_ in their breast milk had lower height-for-age scores (Mahdavi et al. 2010).

Since raw milk in Dagoretti often is sourced from cows there or the surrounding area, the high level of AFM_1_ contamination in raw milk indicated that cows here are exposed to AFB_1_-contaminated feeds; this could be due to the use of commercial feeds which have been shown to be highly contaminated in other places in Kenya and is likely true also in Dagoretti (Senerwa et al. 2016). In addition, it is not uncommon that farmers give their livestock mouldy food leftovers, which could also contribute to high AFM_1_ levels (Kiama et al. 2016). The pasteurised milk may however have arrived from other parts of the country.

A commercial ELISA was used for laboratory analysis, and the protocol and standards provided by manufacturers was used, with no additional reference samples. The method was judged reliable with little variation between duplicates and high *R*^2^ values for the regression of the standards. In the study by Berhanu et al. (under preparation), spiked samples have been used as reference material, with recovery above 70%, but this is lower compared to the study by Ismail et al. (2017) where recovery was between 94 and 100%, using the same kit. The same ELISA has been used by several other studies including to test fermented milk samples (Temamogullari and Kanici 2014; Farah Nadira et al. 2017). Yoghurt was not spiked in this study, but other studies have found the recovery rates of spiked yoghurt samples being lower than milk samples (Kim et al. 2000). This indicates that the results for yoghurt in our study may possibly be underestimating the actual levels.

In conclusion, this study finds that much of milk purchased in urban Nairobi contains AFM_1_ above the recommended levels, but that there are variations both seasonally and from different processors. Urban inhabitants tend to eat more animal-source foods (Rae 1998), and in Kenya, where milk consumption is important for the population, there has been many concerns about the safety, particularly for aflatoxins. While levels of AFM_1_ in milk are still much lower than aflatoxins in crops in Kenya and the toxin is less potent, these results suggest that it might be possible to work with processors to identify measures to reduce the contamination and thus achieve lower levels in the milk sold in Nairobi.

## Abbreviations

AFB_1_: Aflatoxin B_1_
AFM_1_: Aflatoxin M_1_
ELISA: Enzyme-linked immunosorbent assay
KEBS: Kenyan Bureau of Standards
KES: Kenyan Shilling
UHT: Ultra-high temperature
